# Immune Dysfunction Mediated by the ceRNA Regulatory Network in Human Placenta Tissue of Intrahepatic Cholestasis Pregnancy

**DOI:** 10.3389/fimmu.2022.883971

**Published:** 2022-06-24

**Authors:** Yuya Wang, Yan Tang, Xianli Yang, Jie Xu, Yanjie Chen, Jing Xu, Shan Hu, Ping Yi

**Affiliations:** Department of Obstetrics and Gynecology, The Third Affiliated Hospital of Chongqing Medical University, Chongqing, China

**Keywords:** intrahepatic cholestasis of pregnancy, ceRNA, immunity-related molecules, birth weight, placenta weight, bioinformatics analysis

## Abstract

Pregnancy-related intrahepatic cholestasis (ICP) is a serious complication with adverse perinatal outcomes of preterm labor, fetal distress, or stillbirth. As a result, it is important to investigate and identify the potential critical pathogenic mechanisms of ICP. First, we collected the placental tissues from the ICP with placental weight and fetal birth weight loss for the whole transcriptome sequencing. Then we analyzed the differentially expressed (DE) circRNAs (DEcircRNAs) by SRPBM, DElncRNAs by FRKM, DEmiRNAs by TPM, and DEmRNAs by TPM and RSEM. Based on differential expression of term pregnancy placental tissues from pregnancies impacted by ICP (n=7) as compared to gestational aged matched control tissues (n=5), the circ/lncRNA-miRNA-mRNA competitive endogenous RNA (ceRNA) regulatory networks were constructed. The ceRNA regulatory networks covered 3,714 events, including 21 DEmiRNAs, 36 DEcircRNAs, 146 DElncRNAs, and 169 DEmRNAs. According to the functional analysis, ICP complications were linked to the immune system, signal transduction, endocrine system, cell growth and death, and transport and catabolism. Further evidence suggested that the expression of immune-related genes *KLRD1*, *BRAF*, and *NFATC4* might have a potential ceRNA mechanism by individual lncRNA sponging miR372-3p, miR-371a-3p, miR-7851-3p, and miR-449a to control downstream the level of TNF-α, IFN-γ, and IL-10, thereby regulating the pathophysiology of ICP. Furthermore, our results were validated by the qRT-PCR, western blotting and ELISA assays. In conclusion, this study is the first to evaluate placental ceRNA networks in pregnancies affected by ICP, showing alterations in immune regulatory networks which may impact fetal and placental growth. Overall our these data suggest that the ceRNA regulatory network may refine biomarker predictions for developing novel therapeutic approaches in ICP.

## Introduction

The competitive endogenous RNA (ceRNA) hypothesis that protein-coding messenger RNAs (mRNAs) and noncoding RNAs (ncRNAs) crosstalk with and regulate each other by microRNA response elements (MREs) competing for binding to common miRNAs (1, 2). MREs were known as the guide strand, retained within miRNA-induced silencing complex (miRISC), targeted to mRNA with partially complementary sequences (3, 4).ceRNAs serve as endogenous sponges,the competitive inhibitors of miRNA function, showing another novel layer of posttranscriptional regulation (1, 5). Recently, many researchers have focused on the ceRNA interactions which uncover a novel mechanism and play important roles in differentiation (2, 6, 7), cancer (8–11), immune-related diseases (12), cardiovascular diseases (13, 14), neurological diseases (15, 16), etc. Linc-MD1 “sponges” miR-133 and miR-135 to regulate the expression of transcription factors MAML1 and MEF2C, then to governs the time of muscle differentiation in mouse and human myoblasts (2).The lncRNA H19 functions as a ceRNA to sponge miRNA let-7 family leading to an increase in expression of let-7 targets in breast cancer,ovarian cancer and pancreatic cancer (8).Notably, ceRNA also acts crucial role in reproductive health (17), such as fetal and organ development (18), spontaneous abortion (19–21) and eclampsia in pregnancy (22). In pre-eclampsia, circVRK1 acts as a ceRNA to miR-221-3p to regulate PTEN, and further inhibit PI3K/Akt activation, thereby suppressing trophoblast cell migration, invasion and EMT (22). Therefore, most ceRNA interactions between mRNAs are linked to various disease states, but few have been linked to pregnancy-related diseases and pregnancy complications, ICP in particular.

Intrahepatic cholestasis of pregnancy (ICP) is a complication in 0.2-2% of pregnancies, characterized by maternal pruritus and elevated serum bile acids, transaminases, and occasionally, bilirubin (23–25). Its causative mechanism remains unknown, and the studies available are associated with hormonal, immunological, genetic, and environmental factors during pregnancy (26, 27). Ursodeoxycholic acid is controversial as a treatment, though it improves biochemical parameters (23). As a result, there is no effective treatment for ICP (23). ICP has been considered a benign and reversible disease for mothers, but perinatal babies, suffering the severe adverse pregnancy outcomes of fetal distress, spontaneous and iatrogenic preterm birth, and stillbirth (23, 28). Interestingly, recent studies have suggested that women with ICP increased the risk of later hepatobiliary cancer and immune-mediated and cardiovascular diseases (29).

The placenta is a temporary mammalian organ that connects the maternal and fetal circulatory systems. Molecules produced by the placenta contribute to fetal developmental programming and support the maternal organism to cope with the response of pregnancy (30). The placental-associated gene expression alterations may lead to its aberrant function and pregnancy complications (31–34). Therefore, a comprehensive, in-depth, and systematic understanding of the alterations and their associated actions in placental tissues is of great significance for making out the pathogenesis and adverse perinatal outcomes of ICP. Based on the principles of ceRNA regulation in pregnancy-related diseases, the whole transcriptome sequencing of placental tissues of ICP was done at the first time to profile the DEcircRNAs, DElncRNAs, DEmiRNAs, and DEmRNAs, construct the ceRNA regulatory networks, and explore the capability of ceRNA in the process of ICP.

## Material and Methods

### Data Resource

From January 2021 to June 2021, seven ICP pregnant women and five women as normal control (NC) were enrolled and delivered by cesarean section at the Maternity Center of the Third Affiliated Hospital of Chongqing Medical University. The inclusion criteria for the ICP group were: fasting serum total bile acid (TBA) level ≥10 μmol/L; with or without the presence of pruritus; elevated glutamate transaminase (ALT) and alanine transaminase (AST) with unknown causes; and the above symptoms and laboratory parameters disappeared after delivery. The Inclusion criteria for the control group were: no complications or comorbidities of pregnancy; no previous history of preterm birth, macrosomia, or low birth weight babies. Exclusion criteria: the existence of pre-pregnancy liver, biliary and pancreatic diseases, autoimmune diseases, combined hypertension during pregnancy, gestational diabetes or other pregnancy complications, and medical and surgical comorbidities. The indications for this cesarean section were: patients and family’s request or scarred uterus. The study was approved by the Hospital Medical Ethics Committee (202107), and informed consent was followed for each pregnant woman participating in the experiment.

### Sample Collection

Samples were obtained from the villous placenta, mid-way between the chorionic and basal plates, at four different positions within 5 minutes after placental separation during cesarean delivery. These placental tissues were washed with DEPC water to remove residual blood as possible, weighed and then placed into an RNA later solution or empty centrifugal tube and stored at -80°C or in liquid nitrogen.

### RNA-Seq

According to the manufacturer’s instructions, total RNAs were isolated using the RNeasy Plus Universal Mini Kit (Qiagen). High-quality RNA samples (OD260/280 = 1.8~2.2, OD260/230≥2.0, RIN≥8, 28S:18S≥1.0, >10 μg), verified by 2100 Bioanalyzer (Agilent Technologies, Santa Clara, CA, USA) and the ND-2000 (NanoDrop Technologies), were constructed the sequencing library. Total RNAs (5 μg) were obtained following TruSeqTM stranded total RNA Kit from Illumina (San Diego, CA) to prepare for the transcriptome strand library. Firstly, ribosomal RNA (rRNA) was depleted with Ribo-Zero Magnetic kit and then fragmented by fragmentation buffer. Next, the first-stranded cDNAs were synthesized by random hexamer primers. After removing RNA templates, the ds cDNAs were generated with dUTP in place of dTTP. Those ds cDNAs were isolated by AMPure XP beads with a single ‘A’ nucleotide added at 3’ ends of the blunt fragments. Finally, multiple indexing adapters were ligated to the ends of the ds cDNAs. The 200–300 bp cDNA target fragments were selected, amplified, and quantified by TBS380. The RNA-seq library was sequenced by the Illumina HiSeq xten//NovaSeq6000 (2 × 150 bp read length). Additionally, sequencing adapters were ligated to total RNAs (3 μg) with TruseqTM Small RNA sample prep Kit (Illumina, San Diego, CA, USA). The ligated RNAs were transcribed to cDNA, then amplified (12 cycles) for libraries, quantified, and constructed by deep sequencing using Shanghai Majorbio Bio-Pharm Biotechnology Co., Ltd. (Shanghai, China).

### Read Mapping and Transcriptome Assembly

The raw paired-end reads were trimmed and quality controlled by SeqPrep (https://github.com/jstjohn/SeqPrep) and Sickle (https://github.com/najoshi/sickle) with default parameters. Then clean reads of RNA-seq were aligned to the human reference genome with orientation mode using HIASAT (https://ccb.jhu.edu/software/hisat2/index.shtml) software. StringTie (https://ccb.jhu.edu/software/stringtie/index.shtml?t=example) was used to assemble transcripts. Raw counts for annotated genes (protein-coding genes, rRNA, microRNA, LncRNA) in the General Transfer Format (GTF) annotation file was obtained.

### Principal Component Analysis

To reveal the RNA-seq profile of placenta from ICP and normal pregnant women, we performed principal component analysis, and PC1-PC3 was used to correct and distinguish those samples.

### Identification of Differentially Expressed (DE) RNAs

We analyzed differential expressed genes (DEGs) between the ICP and normal pregnant women (as a reference). TPM method was calculated the expression level of each transcript, and RSEM was quantified for gene abundances (http://deweylab.biostat.wisc.edu/rsem/). The *DESeq2/DEGseq/EdgeR* with adjusted *P-*value were used together to determine whether a gene is differentially expressed. If adjusted *P*-value ≤ 0.05 (*DESeq2 or EdgeR*), differential expressed mRNAs (DEmRNAs) with fold change > 2 or < -2, the gene was considered differentially expressed between two groups of samples.

The Coding Potential Calculator (CPC), Coding-Non-Coding index (CNCI), and Coding Potential Accessment Tool (CPAT) were applied to filter transcripts with coding potential. Then according to Pfam HMM, those transcripts with known protein domains were excluded by Pfam Scan. FRKM method was used to calculated the expression level of each lncRNA, and RNAs with |log2FC| >1 and FDR < 0.05 by EdgeR were thought to be significant differently expressed lncRNAs (DElncRNAs).

The CIRI (CircRNA Identifier) tools were used to identified circRNA and eliminate false positive candidates resulting from incorrectly mapped reads of homologous genes or repetitive sequences. Each circRNA’s expression level was calculated by Spliced Reads per Billion Mapping (SRPBM) method. CircRNAs were extracted with |log2FC| >1 and *P*-value < 0.05 by DEseq and to construct the significant differently expressed circRNA set (DEcircRNAs).

Low-quality bases (Sanger base quality of < 20) of the 3’ end and sequencing adapters were removed with the in-house perl scripts and the fastx toolkit software, respectively. All sizes ranging from 18 to 32 nt were eliminated from the initial data set. The non-miRNA sequences (rRNA, tRNA, snoRNA, etc.) were removed by a BLAST search of the Rfam database, version 10.1 (http://rfam.sanger.ac.uk/). The perfectly matched sequences from the BLAST search of the miRbase (version 21.0), were used to count and analyze the known miRNA expression profile. The hairpin structure of miRNA precursor can predict novel miRNA. Each miRNA’s expression was calculated according to the transcripts per million reads (TPM) method. If |log_2_FC| >1 and FDR < 0.05 by DEseq2, the miRNAs were defined as differently expressed miRNAs (DEmiRNAs).

### Gene Ontology (GO) and KEGG Annotation Analysis

To profile gene functions, we performed Gene Ontology Annotation for Gene lists. We performed annotation analysis for GO and KEGG pathways Annotation for Gene lists.

### CeRNA Network

The psRobot was used to predict the lncRNA-miRNA-gene pairs and circRNA-miRNA-gene pairs. The Pearson correlation analysis was used to determine any positive correlations between DEcircRNAs, DEmiRNA, DElncRNAs, and DEmRNA in the ceRNA regulatory network. DElncRNAs targeted DEmRNAs, and interacted miRNAs were deleted from the ceRNA network in the opposite expression pattern between DElncRNAs and the targeted DEmRNAs. Hmisc and complot packages were used to compute and visualize the correlations. Those RNAs with Pearson correlation coefficients greater than 0.5 and *P* < 0.01 were employed. The ceRNA network was constructed by Cytoscape and presented by Sankey plot using the galluvial R package.

### qRT-PCR Verification

According to the manufacturer’s instructions, total RNAs were extracted by TRIzol reagent (Invitrogen) from individuals subjected to ICP patients and controls. The RNA was purified and reverse transcribed to cDNA by PrimeScript RT Reagent Kit (Takara). Finally, qRT-PCR was done with specific primers (**Table S10**) by TB Green Fast qPCR Mix (Takara): 95°C for 30 s, 40 cycles, 95°C for 10 s, 60°C for 30 s. Statistical analyses were carried out using GraphPad Prism software (version 7.0). All *P*-values are two-sided. *P* < 0.05 was considered statistically significant.

### Western Blotting

The placenta tissues were taken from the liquid nitrogen tank and homogenized by grinding the tissue in liquid nitrogen. The homogenate was collected, added to the tissue lysis buffer (P0013, Beyotime) with PMSF and cocktail, and sonicated (10s, 30s, 5-10 cycles) to make the tissue fully lysed. The supernatant was collected by centrifugation (12000 rpm/min, 10-15 min) and set aside at -80°C. The proteins were boiled with 5×SDS loading buffer, resolved by SDS-PAGE, and measured by indicated antibodies and anti-rabbit or anti-mouse secondary antibody conjugated with horseradish peroxidase. Specific bands were visualized by enhanced chemiluminescence (ECL). Antibodies against the following epitopes or proteins were obtained from the indicated suppliers: NFATC4 (ab3447, Abcam), BRAF (20899-1-AP, Proteintech) and GAPDH (60004-1-Ig, Proteintech).

### ELISA

The placenta tissues (removed residual blood) were taken from the liquid nitrogen and were mashed by tissue mashers (10000-15000r/min). And the pre-chilled PBS (0.01M, pH=7.4) with PMSF and cocktail were added to the homogenizer. Then the homogenate was centrifuged at 5000×g for 5-10 min and the supernatant was collected for ELISA. The levels of secreted IL-2, IL-10, IFN-γ, and TNF-α in placenta tissue were detected by ELISA kits IL-2 (mlbio, ml058063, China), IL-10 (mlbio, ml064299, China), IFN-γ (mlbio, ml077386, China), and TNF-α (mlbio, ml077385, China), according to the manufacturer’s instruction.

## Results

### Clinical Data on ICP Placenta

**Figure 1:**
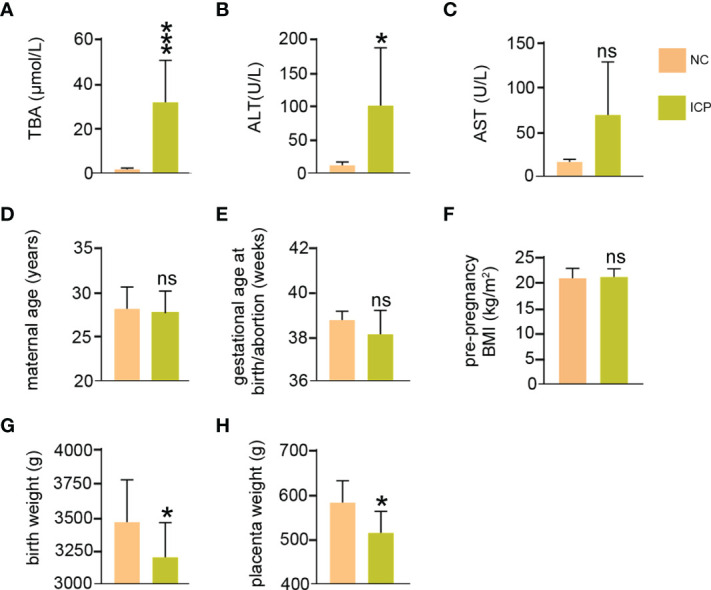
The clinical data of pregnant women with ICP. **(A)** Maximum TBA levels during pregnancy of ICP group and normal group. **(B, C)** Maximum ALT and AST levels during pregnancy. **(D)** Maternal age at the time of termination of pregnancy. **(E)** Gestational age at birth. **(F)** Pre-pregnancy body mass index. **(G)** Fetal weight at birth. **(H)** Placenta weight at the time of termination of pregnancy. ns means not statistically significant *P < 0.05; vs. normal group.

As shown in **Figure 1**, the TBA of ICP was significantly higher than that of NC (*P* < 0.001) (**Figure 1A** and **Table S1**). ALT (*P* < 0.045) and AST (*P* < 0.079) were slightly elevated in the ICP group compared with that in the control group, although there is no statistical significance in AST (**Figures 1B, C** and **Table S1**). No significant statistical difference has been found in the maternal age (y24-30 vs. y25-32), gestational age at birth/abortion (weeks, 37-39.3 vs. 38.3-39.3), and pre-pregnancy BMI (kg/m^2^, 19.5-24.0 vs. 19.2-24.2) between the ICP and NC group (**Figures 1D–F** and **Table S1**). Of note, the birth weight (*P* < 0.036) and placenta weight (*P* < 0.039) of ICP losses significantly compared to that of the NC group (**Figures 1G,H** and **Table S1**). In summary, clinical data showed that the weight loss of the fetus and placenta arose in ICP compared to that in the NC groups.

### RNA-Seq of Human Placenta Tissue

**Figure 2:**
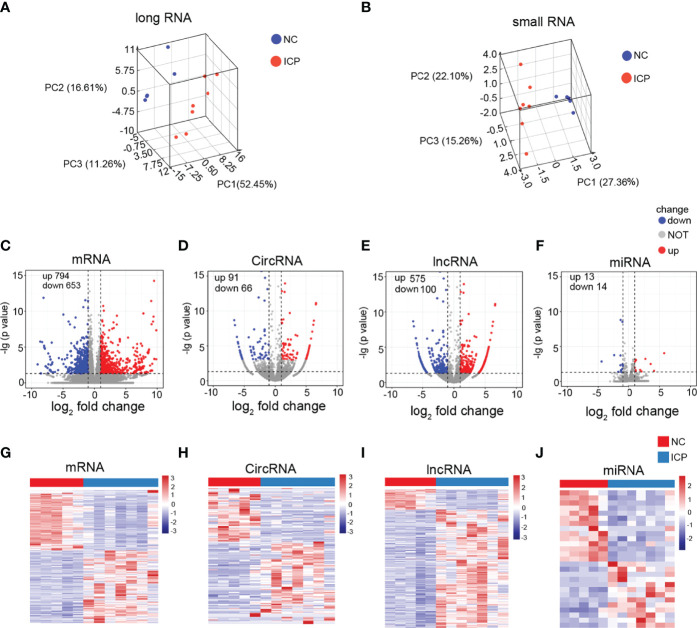
Identification of the DERNAs in placenta tissue of ICP. **(A, B)** Box plots: the distributions of the datasets for long RNAs **(A)** and small RNAs **(B)** of twelve samples. **(C-F)** Volcano plots of the DEmRNAs **(C)**, DEcricRNAs **(D)**, DElncRNAs **(E)**, and DEmiRNAs **(F)** (red, up-regulated; blue, down-regulated). **(G-J)** Heatmaps for DEmRNAs **(G)**, DEcricRNAs **(H)**, DElncRNAs **(I)**, and DEmiRNAs **(J)** (red, up-regulated; green, down-regulated).

RNA-Seq results for twelve tissues (including seven ICP and five normal placenta tissues) were used for the comprehensive analysis. Information and quality of sequencing data are shown in **Table S2**. The reads distribution of 12 samples is shown in **Figures S1-4**. The datasets for the long RNA (circRNAs/mRNAs/mRNAs) and small RNA (miRNA) from ICP and NC groups were distinguished after normalization (**Figures 2A, B**). Total 1447 significant differentially expressed mRNAs (DEmRNAs) (794 upregulated and 653 downregulated), 157 significant DEcircRNAs (91 upregulated and 66 downregulated), 675 DElncRNAs (575 upregulated and 100 downregulated), and 27 significant DEmiRNAs (13 upregulated and 14 downregulated) were displayed in detail on **Tables S3-6**. In addition, DEmRNAs, DElncRNAs, DEcircRNAs, and DEmiRNAs were presented by volcano plots (**Figure 2C**) and heatmaps (**Figure 2D**), respectively.

### Construction of the ceRNA Regulatory Network

**Figure 3:**
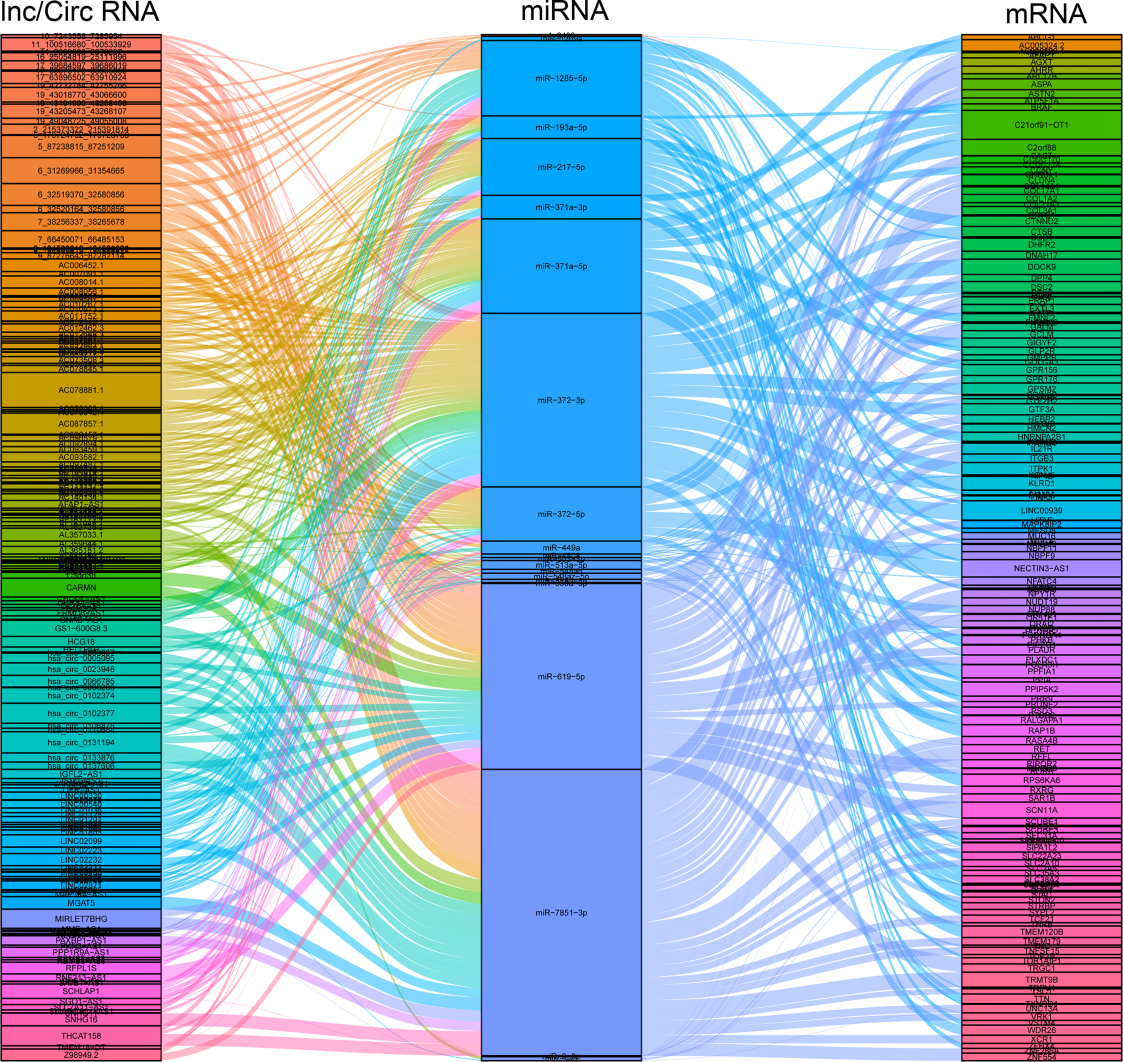
Construction of the circ/lncRNA-miRNA-mRNA ceRNA regulatory network. The ceRNA regulatory network included 21DEmiRNAs, 36 DEcircRNAs, 146 DElncRNAs, and 169 DEmRNAs. ceRNA, competing endogenous RNA; circRNAs, circular RNAs; miRNAs, microRNAs; DE, differentially expressed.

Long non-coding RNA (lncRNA), circular RNA (circRNA), and microRNA (miRNA) play prominent roles in pregnancy-related diseases (35–38). The ceRNA networks, composed of the lnc/circ/miR/mRNA, have been rarely reported in pregnancy-related diseases and pregnancy complications, especially in ICP. As a result, 157 DEcircRNAs, 675 DElncRNAs, 27 DEmiRNAs, and 1447 DEmRNAs were used to construct ceRNA networks to identify and investigate their roles in ICP. The ceRNA events occurred 3714, involving 21DEmiRNAs, 36 DEcircRNAs, 146 DElncRNAs, and 169 DEmRNAs (**Figure 3** and **Table S7**). The candidate ceRNAs might provide a comprehensive and illuminating insight into the molecular mechanisms of ICP.

### Functional Analysis of the DEmRNAs in ceRNA Regulatory Network

**Figure 4:**
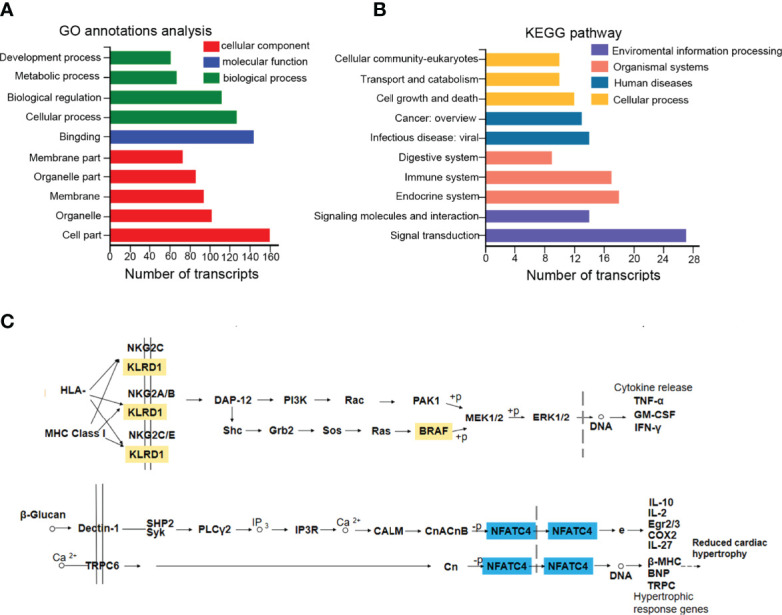
The functional analysis of DEmRNAs in the ceRNA regulatory network. **(A, B)** 169 DEmRNAs were presented in GO and KEGG annotation analysis. **(C)** KEGG annotated diagram of the signaling pathways. Up-regulated labeled in orange and down-regulated labeled in blue.

Those 169 DEmRNAs (57 upregulated and 112 downregulated) from the circRNA/lncRNA-miRNA-mRNA ceRNA regulatory networks were analyzed and conducted with KEGG and GO annotation analysis (**Tables S8-9**). GO annotations showed that the DEmRNAs were mainly involved in cell part, binding, and cellular process to regulate cellular component, molecular function, and biological function (**Figure 4A**). Notably, these mRNAs of KEGG analysis were mostly enriched in signal transduction, endocrine system, immune system, cell growth, and death, as well as transport and catabolism (**Figure 4B**). The signal transduction pathways included MAPK, PI3K-AKT, mTOR, and Wnt signaling pathways, participating in immunomodulation, protein synthesis, and survival (**Table S9**). The endocrine system involved the estrogen signaling pathway, affecting apoptosis, cell adhesion, cell membrane components, and cytoplasmic signaling cascade response (**Table S9**). The immune system caused the changes of cytokines and chemokines (**Table S9**). Cell growth and death mainly caused cellular apoptosis, necroptosis, and ferroptosis (**Table S9**). Moreover, it was of great interest that both *BRAF* and *NFATC4* shared the top 5 transcript collections in GO and KEGG analysis (**Tables S8-9**, colored yellow). BRAF promotes the release of cytokines such as TNFα, GM-CSF, and IFN-γ (map04650), while NFATC4 alters the level of IL2 and IL10 (map04625) (**Figure 4C**). Previous studies have established that elevated TNFα and IFN-γ can damage the fetus and placenta (39, 40). As shown in **Figure 4C**, KLRD1 is the component of the multiple immune complexes, which trigger the immune response. Therefore, the immune dysfunctions mediated by the ceRNA regulatory network (BRAF- and its upstream KLRD1- and NFATC4-dependent ceRNA) offered novel sight and approaches for the progression of ICP.

### The Validation of Differentially Expressed Genes in the ceRNA Network

**Figure 5:**
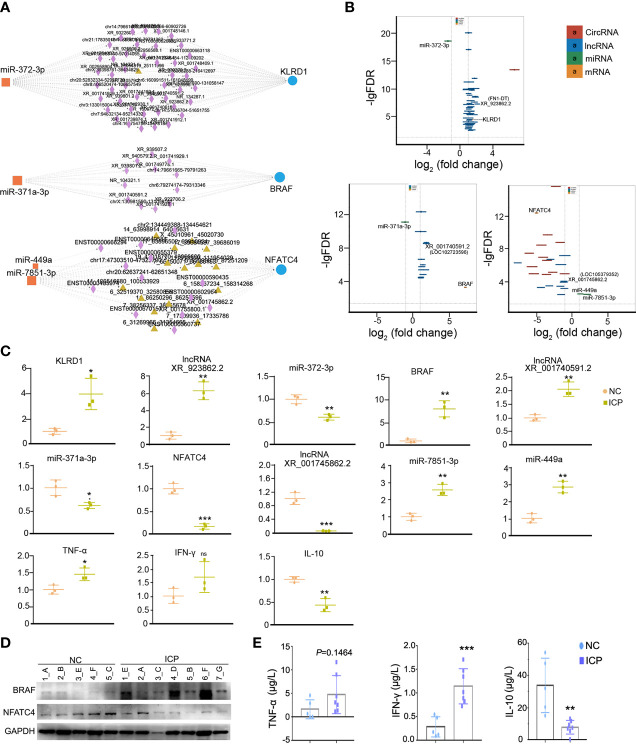
The validation of obviously differentially expressed RNAs in the ceRNA network. **(A)** The ceRNA network of KLRD1, BRAF, and NFATC4 (rectangles, DEmiRNAs; triangles, DEcircRNAs; diamonds, DElncRNAs; Circles, DEmRNAs). **(B)** The fold changes of KLRD1, BRAF, and NFATC4 were involved in ceRNA regulatory network in the ICP group compared with the control group. **(C)** The validation on RNA expression in the ICP group (n=3) compared with the control group (n=3) by qRT-PCR. Each dot represents the average value of one sample in three experimental replicates. **(D)** The protein level of NFATC4 and BRAF in the ICP group compared with the control group. The letters represented the different placenta tissues. **(E)** Comparison of cytokines levels in placenta tissue between ICP and healthy controls. Cytokines concentrations of TNF-α, IFN-γ and IL-10 were compared between ICP patients (n=7) and healthy controls (n=5). Data were shown as mean ± SD. Differences were analyzed by unpaired *t* test. All P-values are two-tailed and significantly different when *P*-value is <0.05. *P<0.05, **P<0.01, ***P<0.001.

The represented ceRNA events were chosen for qRT-PCR validation of placental tissues in clinical specimens. The ceRNA regulatory networks of KLRD1, BRAF, and NFATC4 were illustrated in **Figure 5A**, and their expressions obtained from RNA-seq were shown in **Figure 5B**. The results of qRT-PCR that the expression of lncRNA (XR_923862.2, XR_001740591.2, XR_001745862.1), miRNA (miR372-3p, miR-371a-3p, miR-7851-3p, and miR-449a), mRNA (KLRD1, BRAF, and NFATC4), and the downstream cytokines and chemokines (TNFα, IFN-γ, and IL-10) in the placental tissues (NC n=3, ICP n=3) were consistent with the sequencing data (**Figures 5C**). To further solidify our conclusions, we examined the protein expression levels of NFATC4 and BRAF in placental tissues and found that, consistent with mRNA levels, NFATC4 expression was decreased and BRAF was elevated in ICP placental tissues (n=7) (**Figure 5D**). Besides, the inflammatory factors IL-10 decreased, IFN-γ and TNF-α increased. Also, IL-10 and IFN-γ showed significantly statistical differences in ICP placental tissues (n=7) compared to that in healthy controls (n=5) (**Figure 5E**). Altogether, ceRNA networks were involved in the process and adverse perinatal outcomes of ICP.

## Discussion

Our clinical data provided evidence of the weight loss of the placenta and fetus in ICP compared to normal pregnant women, suggesting that ICP retrained placental development and fetal growth (**Figure 1**). The whole transcriptome sequencing of placental specimens from ICP patients was performed to better investigate the mechanisms of placental growth and fetal development during ICP. A novel aspect of our study was construction of ceRNA regulatory networks from analysis of differential expression libraries of mRNA, circRNA, lncRNA and miRNA in placental tissues from pregnancies affected by maternal ICP (**Figures 2**, **3**). These data supported that the role of ceRNAs (lnc/circRNA-miRNA-mRNA) was one of the major contributors to ICP. Futher, ceRNA serving as the competitive inhibitors of miRNA function, has been well established among various diseases including pregnancy-related disorders *via* celluar models and annimal models. In polycystic ovary syndrome (PCOS), lncRNA MALAT1 reduction could suppress TGFβ signaling through sponging miR-125b and miR-203a in granulosa cells (41). In unexplained recurrent spontaneous abortion, circRNA-DURSA/miR-760/HIST1H2BE axis, lncRNA-HZ04/miR-hz04/BPDE axis, and lncHZ05/miR-hz05/BPDE axis were proved to affect human trophoblast cell proliferation and apoptosis (42–44). In preeclampsia, lnc00511 was functioned as a molecular sponge for miR-29b-3p, antagonizing its ability to repress Cyr61 protein translation (45).

Further, the GO and KEGG analysis evidenced that BRAF and NFATC4 shared the top 5 transcript collections (**Tables S8-9**, colored yellow) and regulated the cytokines mediated immune dysfunction (map04650 and map04625). In the course of normal pregnancy, helper T (TH) cell type 1 cytokines are downregulated and TH type 2 cytokines are upregulated at the maternal-fetal interface, aimed at protecting the fetus from cytotoxic T cell responses which are associated with fetal rejection and pregnancy loss (46, 47). It has been confirmed that ceRNA involved in the differentiation of T cell subtypes, which was a side argument to our conclusion. LncRNA SNHG16/miR-16-5p/SMAD5-regulatory axis potentiates TGF-β1/SMAD5 pathway activation, thus inducing CD73 expression in Vδ1 T cells in breast cancer-derived exosomal (48). LncITSN1-2 has been demonstrated that it promotes IBD CD4+ T cell activation, proliferation, and Th1/Th17 cell differentiation by serving as a ceRNA for IL-23R *via* sponging miR-125a in inflammatory bowel disease (IBD) (49). Additionally, KLRD1 and BRAF in our study ascend the TH1-type cytokines such as TNF-α and IFN-γ (**Figures 4**, **5**), and NFATC4 rose TH2-type cytokine IL-10 (**Figures 4**, **5**). That would upset the balance between TH2 and TH1, tending to evolve into TH1 cytokine profiles, which may be potentially harmful in pregnancy. The inability of the mother to switch from TH1 to TH2 cytokine profiles at the fetal-maternal interface has been proposed as one of the primary causes of miscarriage, intrauterine growth restriction and preeclampsia. The TH1 (IFN-γ, TNF-α, and IL-12) cytokines are detrimental to pregnancy, may even cause fetal loss, and whereas TH2 (IL-4 and IL-10) cytokines are protective to pregnancy (50–52). Excess TNF-α promotes trophoblast apoptosis and damages the placenta directly (39). IFN-γ has been rendered bile acid secretion decrease, trophoblast apoptosis, and placental damage (40). IL-10 were identified involvement of the transplacental immune regulation during pregnancy. It has been demonstrated that IL-10 may influence Treg cell homeostasis through its effect on Treg cell Bcl-2 expression both in humans and mice and support the homeostatic and “uterine tolerance” (53, 54). IL-10 contributes to placental growth and remodeling since IL-10^-/-^ mice exhibited placental damage and maternal blood sinus increase (55). Besides, treatment with IL-4 and IL-10 could rescue the adverse effects on placental dysplasia and fetal loss of targeting Tim-3 and CTLA-4 on the pregnancy outcome (56). All above suggested that the effects of ceRNA-induced inflammatory and immune factors were consistent with our clinical profile of placenta and fetus weight loss (**Figure 1**) and the ceRNA causative network (**Figure 5**). In a word, the ceRNA regulatory network mediated the immune dysfunction in human placenta tissue of ICP may restrain fetal development and placental growth and refine biomarker predictions for developing novel therapeutic approaches in ICP.

In addition, a recent study that ICP pregnant women easily take place liver cancer and immune-mediated cardiovascular disease complications (29) made the concept controversial that ICP is a reversible and benign disease for pregnant women. Notably, our result provides new evidence for possible cardiovascular complications in pregnant women with ICP, that NFATC4 may inhibit the expression on downstream molecules of lower cardiac hypertrophy such as β-MHC, BNP, and TRPC (**Figure 4C**). Some studies have reported that ceRNA networks participate in human dilated cardiomyopathy (57).

The limitation of this study was the inability to confirm the ceRNA mechanism because of a lack of cellular models. The ceRNA is known that circRNA and lncRNA can compete to sponge miRNA *via* miRNA response elements (MREs), reversing the gene silencing. It’s hard to identify the MRE that lnc/circRNAs (XR_923862.2, XR_001740591.2, XR_001745862.1) binding to miR-372-3p, miR-371a-3p, miR-7851-3p, and miR-449a. A further limitation of this study was the small samples, the bias that might occur during enrolment into the case series. More samples and experimental validations of these results were needed for more comprehensive analysis and in-depth studies.

In summary, we first evaluated that placental ceRNA networks in pregnancies affected by ICP, showing alterations in immune regulatory networks which may impact fetal and placental growth. In our study, it was found that lncRNA XR_001740591.2/miR-371a-3p/BRAF axis and lncRNA XR_001745862.1/miR-7851-3p, miR-449a/NFATC4 axises most probably caused the restriction of placental and fetal growth.Overall the ceRNA regulatory network may refine biomarker predictions for developing novel therapeutic approaches in ICP.

## Data Availability Statement

The data presented in the study are deposited and accessible through “https://www.ncbi.nlm.nih.gov/sra/PRJNA846869”, accession number PRJNA846869.

## Ethics Statement

The studies involving human participants were reviewed and approved by Ethics Committee of the Chongqing Medical University. The patients/participants provided their written informed consent to participate in this study.

## Author Contributions

YW and YT conducted the data mining and drafted the manuscript, YT and XY prepared the clinical samples, YW and JX conducted bioinformatics analyses, YC did the PCR validation, SH and PY integrated all efforts. JX, YW and YT performed the assays according to the reviewers’ suggestions, revised and responsed the comments. All authors contributed to the article and approved the submitted version.

## Funding

This study was supported by the Chongqing Science and Health Joint Medical Research Project (2019ZDXM030), Science and Technology Research Project of Chongqing Municipal Education Commission (KJQN201900434) to JX, the Natural Science Foundation of Chongqing, China (No. cstc2021jcyj-bsh0055) to YW, and Program for Youth Innovation in Future Medicine, Chongqing Medical University in 2021 (No. W0058) to PY, JX and YW.

## Conflict of Interest

The authors declare that the research was conducted in the absence of any commercial or financial relationships that could be construed as a potential conflict of interest.

## Publisher’s Note

All claims expressed in this article are solely those of the authors and do not necessarily represent those of their affiliated organizations, or those of the publisher, the editors and the reviewers. Any product that may be evaluated in this article, or claim that may be made by its manufacturer, is not guaranteed or endorsed by the publisher.
