# Supplementary material for: Immune Dysfunction Mediated by the ceRNA Regulatory Network in Human Placenta Tissue of Intrahepatic Cholestasis Pregnancy
Source: Front Immunol. 2022 Jun 24;13:883971. doi: 10.3389/fimmu.2022.883971 (PMC9263217; doi:10.3389/fimmu.2022.883971)
Supplement: Supplementary file 1 [file DataSheet_1.zip › supplementary Table/Supplementary Table 1..docx]

**Supplementary Table 1 Clinical information of the enrolled population.**

| Group | Sample size (n) | Maternal age (years) | Gestational age at birth/abortion  (days) | Gestational age at birth/abortion  (weeks) | TBA (μmol/L) | Birth weight (g) | Placenta weight (g) | Fetal  gender (M/F) | Pre-pregnancy BMI (kg/m^2^) | Vaginal/CS delivery |
| --- | --- | --- | --- | --- | --- | --- | --- | --- | --- | --- |
| ICP | 7 | 27.6 | 266 | 38.1 | 32.1 | 3207 | 516.7 | 3/4 | 21.1 | 0/7 |
|  |  | (24-30) | (259-275) | (37-39.3) | (13.6-57.9) | (2750-3480) | (430-575) |  | (19.5-24.0) |  |
| NC | 5 | 28.2 | 271 | 38.8 | 2.1 | 3469 | 584.0 | 2/3 | 20.9 | 0/5 |
|  |  | (25-32) | (268-275) | (38.3-39.3) | (1.6-3.1) | (3100-3880) | (525-645) |  | (19.2-24.2) |  |

**Clinical information of the enrolled population in detail.**

| name | age | Gestational age at birth/abortion  (weeks+days) | CS delivery | Birth weight (g) | Placenta weight (g) | Fetal  gender (M/F) | TBA (μmol/L) | ALT(U/L) | AST(U/L) | Pre-pregnancy BMI (kg/m^2^) |
| --- | --- | --- | --- | --- | --- | --- | --- | --- | --- | --- |
| ICP-A | 29 | 38+4 | Yes | 3480 | 560 | Male | 13.9 | 67 | 40 | 24.03 |
| ICP-B | 24 | 39+2 | Yes | 3460 | 575 | Female | 25.0 | 20 | 13 | 21.64 |
| ICP-C | 31 | 37 | Yes | 3100 | 516 | Female | 57.9 | 215 | 183 | 22.08 |
| ICP-D | 25 | 39+2 | Yes | 3050 | 510 | Female | 13.6 | 32 | 36 | 19.5 |
| ICP-E | 30 | 37+1 | Yes | 3180 | 490 | Male | 54.7 | 55 | 58 | 20.27 |
| ICP-F | 28 | 31 | Yes | 2750 | 430 | Male | 43.2 | 230 | 115 | 19.8 |
| ICP-G | 27 | 38+4 | Yes | 3430 | 536 | Female | 16.3 | 94 | 41 | 20.31 |
| NC-A | 32 | 39 | Yes | 3750 | 620 | Male | 2.4 | 20 | 17 | 24.15 |
| NC-B | 27 | 39+2 | Yes | 3100 | 525 | Female | 1.6 | 8 | 16 | 20.60 |
| NC--C | 25 | 38+4 | Yes | 3880 | 645 | Male | 2.0 | 9 | 15 | 19.23 |
| NC-E | 29 | 38+6 | Yes | 3235 | 550 | Female | 3.1 | 16 | 22 | 19.65 |
| NC-F | 28 | 38+2 | Yes | 3380 | 580 | Female | 1.6 | 11 | 15 | 20.63 |
