# Supplementary material for: Immune Dysfunction Mediated by the ceRNA Regulatory Network in Human Placenta Tissue of Intrahepatic Cholestasis Pregnancy
Source: Front Immunol. 2022 Jun 24;13:883971. doi: 10.3389/fimmu.2022.883971 (PMC9263217; doi:10.3389/fimmu.2022.883971)
Supplement: Supplementary file 1 [file DataSheet_1.zip › supplementary Table/Supplementary Table 10.docx]

**Supplementary Table 10. Primers in this study.**

| Name | primer |  |
| --- | --- | --- |
| IL-10 | Forward: CGCTAGAACCAAGCTGTCCT | |
|  | Reverse:CGCCTTGATGTCTGGGTCTT | |
| TNFα | Forward:AGAACTCACTGGGGCCTACA | |
|  | Reverse: GCTCCGTGTCTCAAGGAAGT | |
| IFN-γ | Forward:ATGGTTGTCCTGCCTGCAAT | |
|  | Reverse:CTTGCTTAGGTTGGCTGCCT | |
| KLRD1 | Forward:AGCCAGCATTTACTCCAGGAC | |
|  | Reverse:GAGCCATTCTCCCACAACCA | |
| BRAF | Forward:AAAAAGGCTGACACCAAGCTG | |
|  | Reverse:TGGGAGGAGTCCCAAAAGGA | |
| NFATC4 | Forward: CCGTTTAGTTGCTGGGATGG | |
|  | Reverse:GACCTGGACTGGAGTTTCCG | |
| miR372-3p | Forward:GTGGG CCTCA AATGT GGAG | |
|  | Reverse:GTGACGCTCAAATGTCGCA | |
| miR-371a-3p | Forward:CAAACTGTGGGGGCAC | |
|  | Reverse:GTAACACTCAAAAGATGG | |
| miR-7851-3p | Forward:CCTCATCCTCCCAAGTAGCTG | |
|  | Reverse:TGGCTCACTGTCGCCTCGAC | |
| miR-449a | Forward:CTGTGTGTGATGAGCTGG | |
|  | Reverse:TGTATATGCAATAAGACAGC | |
| XR_923862.2  (FN1-DT) | Forward:GGTGGTAGTGTTTGAGGAC | |
|  | Reverse:CCGCCCTGGGACTGAAAAG | |
| XR_001740591.2 (LOC102723596) | Forward:GTGCCTTGAATGCTGC | |
|  | Reverse:GTCATAAGAGATGTGGCC | |
| XR_001745862.1  (LOC105379352) | Forward:GCTCGGCGAACCTTCTGCC | |
|  | Reverse:GACTGGAGATGTAGGTGAAG | |
