# Supplementary figures and images for: Immune Dysfunction Mediated by the ceRNA Regulatory Network in Human Placenta Tissue of Intrahepatic Cholestasis Pregnancy
Source: Front Immunol. 2022 Jun 24;13:883971. doi: 10.3389/fimmu.2022.883971 (PMC9263217; doi:10.3389/fimmu.2022.883971)

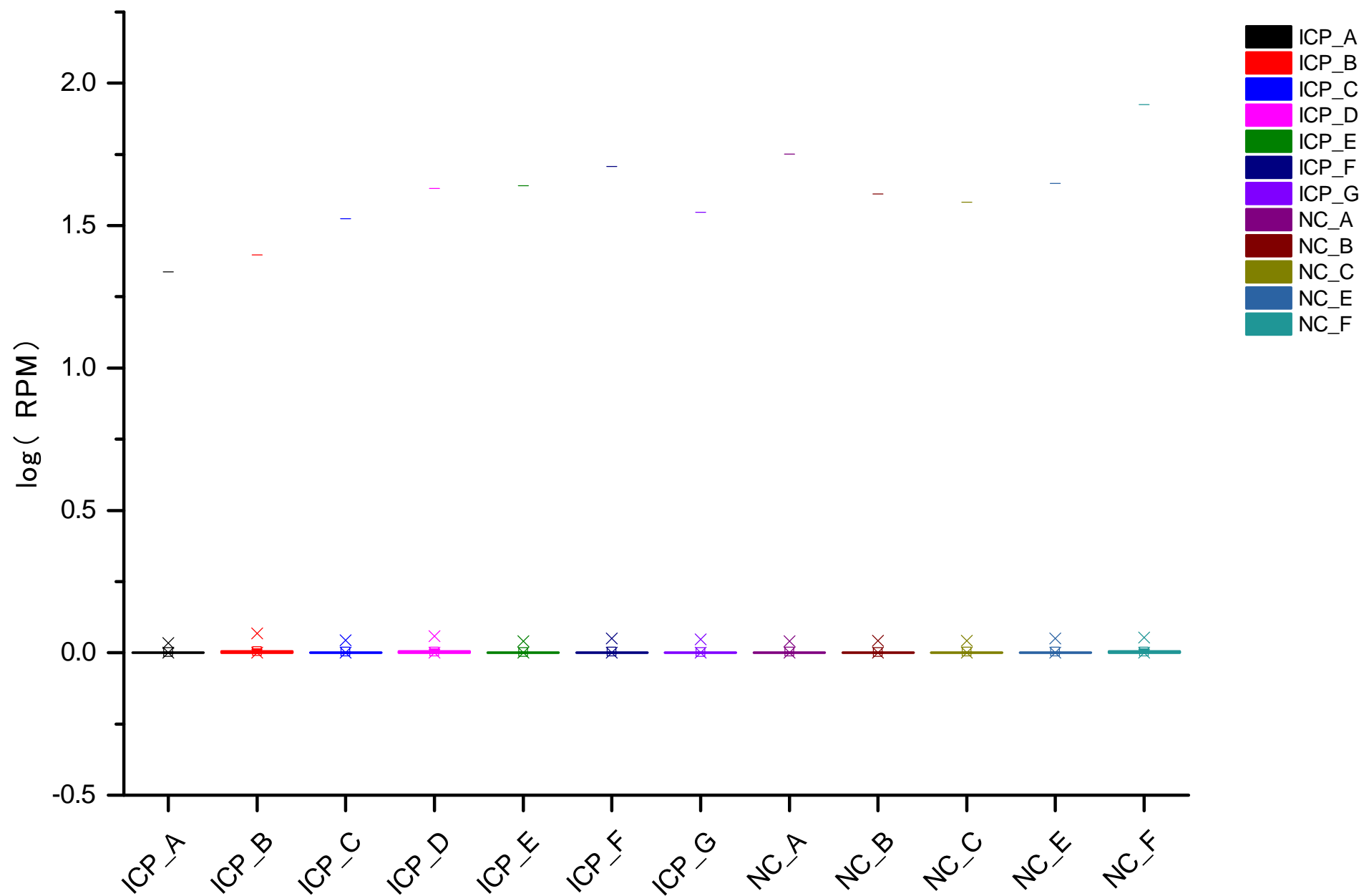

Supplement: Supplementary file 1 [file DataSheet_1.zip › supplementary Figure/Supplementary Figure 1 expression distribution of circRNA.pdf]

Expression Distribution

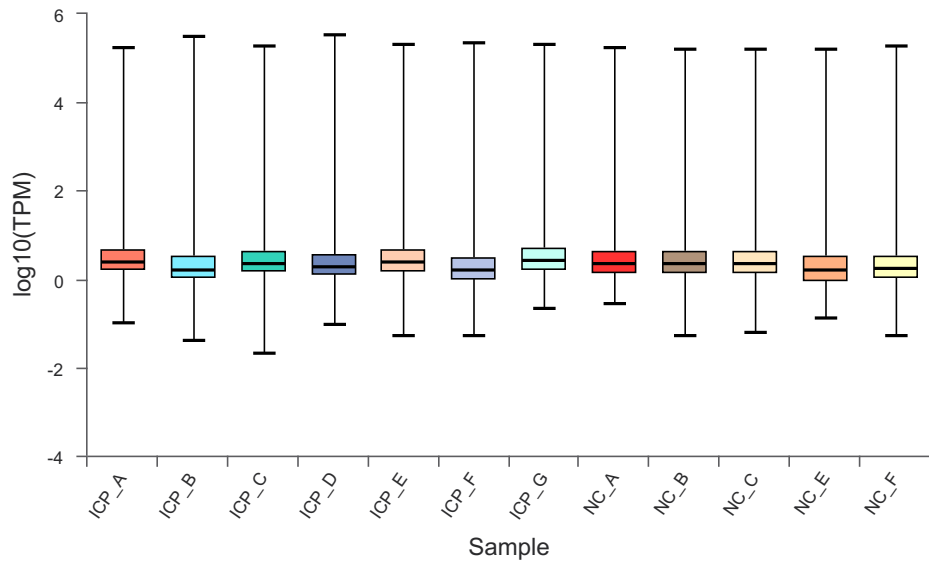

Supplement: Supplementary file 1 [file DataSheet_1.zip › supplementary Figure/Supplementary Figure 2 expression distribution of lncRNA.pdf]

Expression Distribution

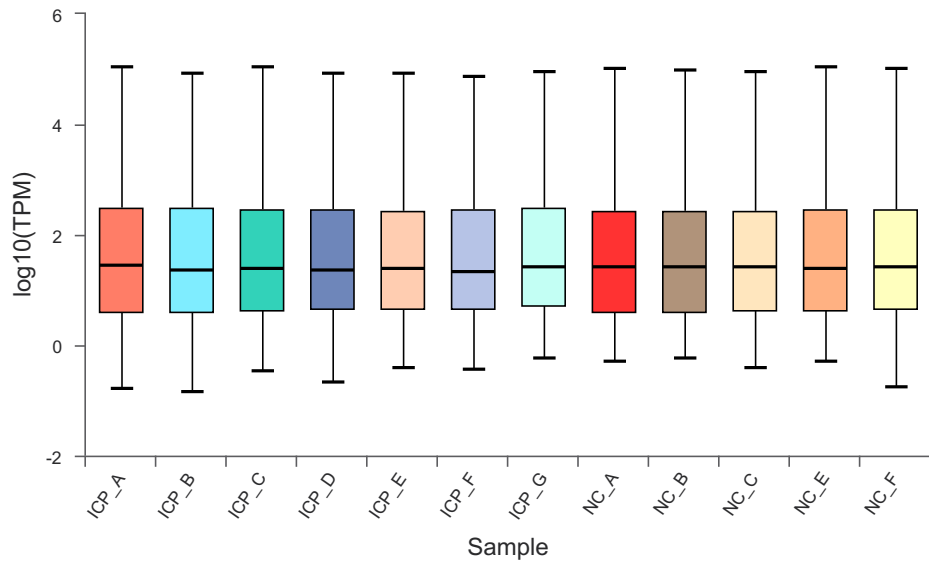

Supplement: Supplementary file 1 [file DataSheet_1.zip › supplementary Figure/Supplementary Figure 3 expression distribution of miRNA.pdf]

Expression Distribution

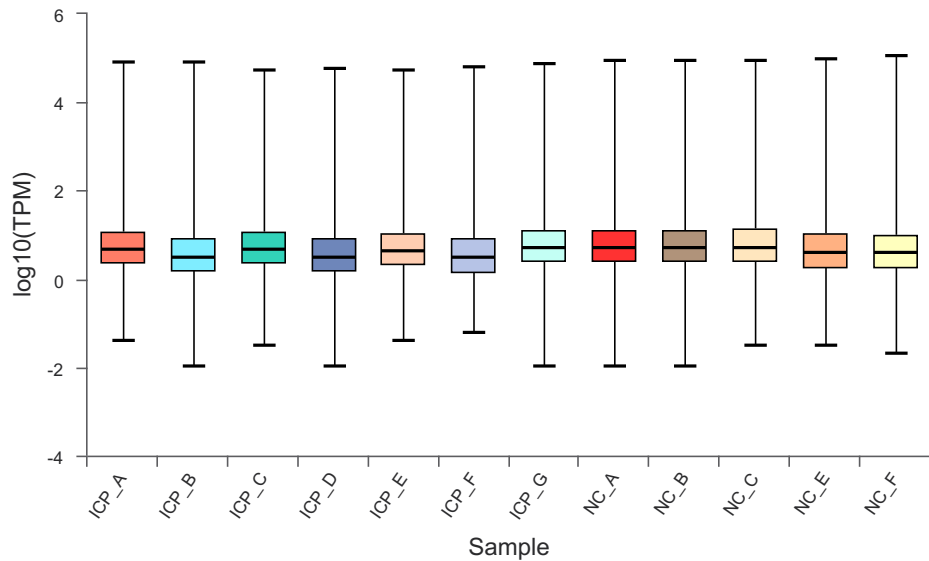

Supplement: Supplementary file 1 [file DataSheet_1.zip › supplementary Figure/Supplementary Figure 4 expression distribution of mRNA.pdf]
